# Differences in income-related health inequalities in Brazil and the USA: a comparative cross-sectional study

**DOI:** 10.3389/fpubh.2026.1917651

**Published:** 2026-10-13

**Authors:** Stefano Zapperi, José S. Andrade, Humberto A. Carmona, Antonio S. Lima Neto, Caterina A. M. La Porta

**Affiliations:** 1 Center for Complexity and Biosystems, Department of Physics, University of Milan, Milan, Italy; 2 CNR - Consiglio Nazionale delle Ricerche, Istituto di Chimica della Materia Condensata e di Tecnologie per l'Energia, Milan, Italy; 3 Departamento de Física, Universidade Federal do Ceará, Fortaleza, Ceará, Brazil; 4 Escola de Saúde Pública do Ceará, Fortaleza, Ceará, Brazil; 5 Center of Health Sciences, University of Fortaleza, Fortaleza, Ceará, Brazil; 6 Secretaria de Saúde do Estado do Ceará, Fortaleza, Ceará, Brazil; 7 Center for Complexity and Biosystems, Department of Environmental Science and Policy, University of Milan, Milan, Italy; 8 UOC Maxillo-Facial Surgery and Dentistry, Fondazione IRCCS Ca' Granda, Ospedale Maggiore Policlinico di Milano, Milan, Italy

**Keywords:** Brazil, health inequality, healthcare, income, obesity, psychiatric conditions, socioeconomic status, USA

## Abstract

**Introduction:**

Health inequality is a persistent, policy-relevant problem shaped by multiple socioeconomic factors, among which income and healthcare policy are especially prominent and often vary substantially between countries. The goal of the present study is to quantify the relationship between income and health in two countries with different healthcare systems, Brazil and the USA.

**Methods:**

We compared health conditions in relation to income in Brazil and the USA through cross-sectional, nationally representative, household-based surveys using data from the Pesquisa Nacional de Saúde (PNS) for Brazil and the Panel Study of Income Dynamics (PSID) for the USA collected in 2019. We used survey-weighted logistic regression with cluster-robust standard errors, adjusted for urban/rural residence, sex, education, and age.

**Results:**

Both countries showed income-related health inequality, with lower-income groups generally reporting worse self-rated health. A formal test of the country-by-income interaction showed a significantly steeper income gradient in the USA than in Brazil for diabetes and obesity and a significant reversal in the direction of the association for psychiatric conditions, with higher income associated with higher prevalence in Brazil but lower prevalence in the USA. No significant cross-country difference was found for hypertension, self-rated health, or cancer diagnosis. Geographically, a north-south divide in inequality is apparent in Brazil, while income disparities are less uniform in the USA. States with a larger share of low-income people experience worse health conditions in the USA, while this effect is less visible in Brazil.

**Discussion:**

Taken together, income-related health inequality is more pronounced in the USA than in Brazil for several, though not all, of the conditions examined. This pattern may be associated with differences in healthcare systems and lifestyles between the two countries, although the cross-sectional design does not permit causal inference.

## 1 Introduction

Health inequality is a persistent global issue reflecting broader societal disparities in income, education, and social status, creating significant variations in health outcomes among different population groups (1, 2). Notably, a strong correlation is often observed between socioeconomic conditions and health, leading to a disproportionate burden of disease and reduced life expectancy for marginalized communities (3, 4). Understanding the multifaceted nature of health inequality and its underlying drivers is crucial for designing effective policies and interventions aimed at promoting health equity (5). A useful tool to study income-related health inequalities comes from comparative analysis of data from different countries. For instance, an international comparative study of inequalities in risk factors for shorter life expectancy across 15 European countries identified low income as one of the quantitatively important entry points for policies to reduce educational inequalities in life expectancy in most European countries (6). Furthermore, from a comparative study of 184 countries it was argues that income inequality effects on health status depend on the country's income level (7).

This study aims to compare the magnitude and direction of income-related differences across six health outcomes in Brazil and the USA and to examine the geographical distribution of these differences within each country using nationally representative survey data from 2019. Examining health inequality across different countries provides a comparative perspective on how different policy approaches, socioeconomic factors, and healthcare systems influence the relation between income and health. The healthcare systems in the two countries have a fundamental difference: Brazil has a universal, publicly funded system providing free healthcare for all residents and visitors, while the USA has a hybrid system in which private insurance dominates, supported by public programs for older adults (Medicare) and low-income groups (Medicaid), but without universal coverage. It is known that Brazil exhibits significantly higher levels of income inequality and lower intergenerational mobility when compared to developed countries, including the USA (8). In Brazil, the top 10% of the population holds 56% of the country's income, while the bottom 50% holds only 9% of it. In the USA, one of the most unequal among the developed countries, the shares are 48 and 10%, respectively. When considering wealth inequality, in Brazil the 10% richest individuals hold 80% of the country's wealth while the bottom 50% holds only 0.5% of it. In the USA these percentages are 70 and 1.5%, respectively (9).

Inequality in Brazil has remained persistently high despite periods of economic growth and are found to exist not just across income groups (10), but also across its geographical regions (11). Regions in the Southeast and South of Brazil generally exhibit higher socioeconomic development and better healthcare provision compared to those in the North and Northeast, highlighting regional disparities that have characterized Brazil during its history (12). Among the factors believed to contribute to Brazilian income inequality, one can mention educational disparities, gender and racial wage gaps, and uneven distribution of formal employment opportunities (13). Furthermore, access to quality healthcare and educational opportunities vary widely, contributing to divergent socioeconomic outcomes (14). Government transfer programs like Bolsa Família and rural pensions have mitigated income inequality, particularly in the short run (13). In the USA, on the other hand, wealth and income inequalities have been increasing in the past decades (15) and are associated to larger health disparities when compared with other developed countries (16). Unlike Brazil, where inequality is strongly patterned by geographic region, income inequality in the USA is more closely tied to differential access to employment-based health insurance and to the presence or absence of state-level Medicaid expansion, which together shape how income translates into realized healthcare access (17). The persistence of a substantial uninsured population in the USA despite its high overall wealth (17) stands in contrast to Brazil's constitutionally guaranteed universal coverage (18), underscoring that the two countries' income-inequality profiles are embedded in very different institutional contexts for converting income into health.

In what follows, we present a comparative analysis of the relation between income and health in Brazil and the USA, based on national surveys from both countries. We study six health outcomes—self-rated health, hypertension, diabetes, obesity, cancer diagnosis, and psychiatric conditions—following the recommendation proposed by Link and Phelan (19) to best characterize the impact of socio-economic status (SES) on health. We then explore how the relations between income and health are geographically distributed within each country. We focus most of our analysis on the year 2019, since this is the most recent year for which Brazilian data are available, but we also complement this individual-level analysis with a trend analysis of a few aggregate population-health indicators (life expectancy and child/infant mortality) for previous years, providing longer-term context for the evolution of health conditions in the two countries against which the 2019 cross-sectional comparison can be situated. Comparing Brazil and the USA allows us to examine whether national income inequality corresponds to the magnitude of individual-level income-health gradients and to consider whether institutional differences, including healthcare-system design and social protection, may help explain the observed patterns. Given the contrasting healthcare systems of the two countries, we hypothesized that income-related gradients in health would generally be weaker in Brazil than in the USA, particularly for chronic conditions whose diagnosis and management depend on sustained access to healthcare. For conditions less directly influenced by healthcare access, we expected smaller cross-country differences.

## 2 Methods

### 2.1 Data

Data on income and health for Brazil for the year 2019 are available through the National Health Survey, or Pesquisa Nacional da Saúde (PNS), a study coordinated by the Brazilian Institute of Geography and Statistics (IBGE) (last accessed on 29/07/2024 from https://www.ibge.gov.br/). The survey involves a multi-stage probabilistic sample of Brazilian adults aged 18 and older, carried out in 2019 and aimed at representing the whole country. Data were obtained from a household questionnaire that collected information on socioeconomic conditions and chronic diseases and lifestyle for *n* = 293, 726 individuals. To study income and health in the USA, we downloaded data from the Panel Study of Income Dynamics (PSID) (20), a public use dataset, produced and distributed by the Survey Research Center, Institute for Social Research, University of Michigan, Ann Arbor, MI (last accessed on 22/08/2024 from https://psidonline.isr.umich.edu/). PSID is a longitudinal panel survey of American families, measuring economic, social, and health factors over the life course of families over multiple generations; for the present study we used the single cross-sectional wave of PSID collected in 2019, with information on *n* = 14, 410 individuals. Statistics on health insurance status and out-of-pocket healthcare expenditure for the USA were obtained from the 2019 National Health Interview Survey (NHIS) (last accessed on 3/12/2024 at https://www.cdc.gov/nchs/nhis/documentation/2019-nhis.html). Notice that sample sizes differ greatly between the surveys for Brazil and the USA, but both were large enough to obtain a large number of statistically significant comparisons, as shown below.

Aggregated statistical series for life expectancy were obtained from the World Bank (WB) database (last accessed on 10/08/2024 at https://data.worldbank.org/). Finally, data on child and infant mortality were obtained from the UN Inter-agency Group for Child Mortality Estimation (last accessed on 02/08/2024 at https://childmortality.org/). These population-level trend indicators are not used in the income-quintile comparisons that form the core individual-level analysis of this study; they are included instead to provide longer-term context on the overall trajectory of population health in the two countries, against which the 2019 cross-sectional income-health comparisons can be situated.

### 2.2 Variables

We identified and selected a set of survey variables that were comparable in PNS and PSID. We used household income, rather than an asset- or wealth-based measure of socioeconomic position, as our primary indicator because it is the socioeconomic variable most directly and comparably harmonized between PNS and PSID. Both surveys collect detailed income information that can be converted to a per-capita household measure using a consistent methodology in each country. We acknowledge that income is more temporally variable than wealth-based measures of socioeconomic position and note this as a limitation in the Discussion. Income in Brazil was quantified considering the sum of formal and informal reported gross income per month. The total household income divided by the number of family members was then assigned to each individual. Informal income is more prevalent in Brazil than in the USA. The Brazilian Institute of Geography and Statistics (IBGE) has been measuring informal income for decades with measures that are methodologically robust and internationally aligned, with published sampling errors and transparent revisions. Under-reporting is unlikely to materially alter who falls in the bottom or top income quintiles since informal earnings are mostly concentrated at the bottom. For the USA, we assigned to each individual (head and spouse) the net family income divided by the number of family members. The difference between gross and net income in the two surveys had no consequences for the present study because we based our analysis on income quintiles and the upper quintile boundary in our data (BRL 2,000 per month, approximately USD 500 at 2019 exchange rates) was slightly above the tax exemption boundary for the year 2019, indicating that including taxes could only have a marginal effect on the quintile quantification. This result is in line with the literature consistently showing that around 80% of households pay no income taxes in Brazil (21). Throughout, comparisons are expressed relative to the bottom income quintile, rural residence, female sex, and less than 9 years of completed education as reference categories (see Section 2.4).

The primary health outcomes of this study are six binary indicators, selected to capture a range of pathological conditions and risk factors relevant to the fundamental-cause framework of Link and Phelan (19): self-rated health, hypertension, diabetes, obesity, cancer diagnosis, and psychiatric conditions. Self-reported health conditions were recorded in both surveys on a scale between 1 and 5. We associated the lowest two grades (poor/fair in the PSID and bad/very bad in the PNS) with poor health condition.

Each outcome is examined within an age range chosen on epidemiological grounds. For self-rated health, we restricted the analysis to the working-age population (18–50) in order to isolate the burden of poor self-rated health status from the effects of the age-associated chronic conditions examined separately below. For hypertension, diabetes, and cancer diagnosis, we restricted the analysis to individuals aged 60 or more, reflecting the ages at which these conditions are most prevalent and most reliably diagnosed in both surveys (see Figures 1e, f, h). No age restriction was applied to psychiatric conditions or obesity, as both are relevant across the adult lifespan. We note that, as a consequence of this scheme, individuals aged 51–59 are included in the psychiatric-condition and obesity analyses but are not covered by either the self-rated-health model (restricted to 18–50) or the three chronic-disease models (restricted to 60 or more); this reflects our aim of contrasting a young, working-age population with an older population most affected by chronic disease, rather than a specific epidemiological threshold for excluding this age band, and we note it as a limitation of the age-stratification scheme in the Discussion.

**Figure 1:**
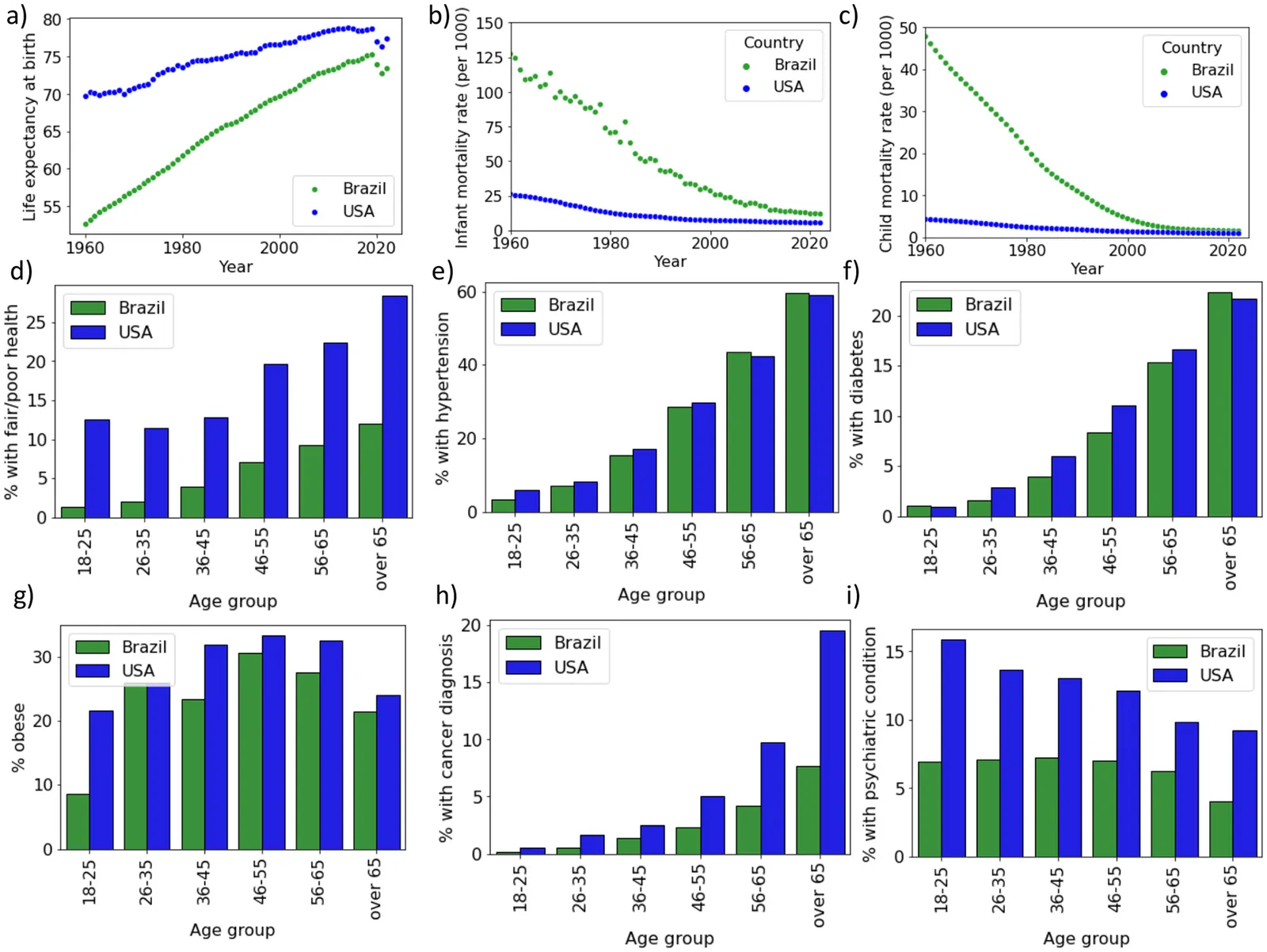
A comparison of health indicators for USA and Brazil. **(a)** Life expectancy at birth, **(b)** infant mortality and **(c)** child mortality from 1960 to 2022. Percentage of the population d) with fair/poor health, **(e)** with hypertension, **(f)** obese, **(g)** with diabetes, **(h)** with a cancer diagnosis, **(i)** with a psychiatric condition for different age groups in the two countries.

Finally, we considered obesity using survey data for self-reported height and weight for the USA, and both self-reported and measured height and weight for Brazil. We then calculated the body-mass index (BMI) of each individual from weight and height. Obesity was defined as a BMI larger than 30 [World Health Organization classification (22)].

For the healthcare-reliance analysis reported in Section 3.4 (Reliance on healthcare across income groups), we used the following variables. For Brazil, PNS records, for each individual, whether they were attended by the Sistema Único de Saúde (SUS) in the 2 weeks preceding the interview, whether they usually seek care through the public health system, whether they have a private health plan or insurance, and household out-of-pocket (OOP) spending on medicines and consultations; we report these indicators by income quintile nationally and by macro-region (Supplementary Tables S7–S9, S11). For the USA, insurance-coverage status (private health insurance, Medicare, Medicare supplement, Medicaid, CHIP, military-related coverage, Indian Health Service, state-sponsored or other government programs, or uninsured) and out-of-pocket healthcare expenditure were both obtained from the 2019 NHIS and are reported by income quintile (Supplementary Tables S5, S6, S10). Because NHIS and PSID are separate surveys, income quintile for the NHIS-based insurance and OOP analyses was defined within the NHIS sample using the same quintile procedure described in Section 2.3, rather than merged at the individual level with PSID. OOP expenditure was expressed as a fraction of household income and is reported by income quintile and by region/census division for both countries (Supplementary Tables S10, S11). Unlike the six health outcomes described above, the healthcare-reliance and OOP indicators are reported as weighted descriptive percentages (with 95% confidence intervals) by income quintile, rather than as adjusted logistic regression odds ratios, since the goal of this analysis is to characterize the pattern of healthcare reliance and financial burden across income groups rather than to test for a covariate-adjusted association.

### 2.3 Calculation of income quintiles

In our analysis, we classified each individual in the survey according to the quintile interval to which it belongs within the income distribution (16, 23). Given the income *Y* whose cumulative distribution is *F*(*Y*), we defined quintiles *Y*_*q*_ as *F*(*Y*_*q*_) = *q*/5 for *q* = 0, ..., 4. The quintile intervals were then defined as [*Y*_0_, *Y*_1_], ... [*Y*_3_, *Y*_4_], and [*Y*_4_, ∞]. In practice, we sorted individuals according to their income and divided them into 5 intervals of equal population share. Sampling weights are present in both surveys and account for population representation. We restricted the analysis to the top and bottom quintiles by design, as our aim was specifically to characterize the health gap between the extremes of the income distribution in each country—the comparison most directly relevant to our central research question—rather than to estimate a dose-response gradient across the full income distribution, which we consider a distinct question outside the scope of the present study.

### 2.4 Statistical analysis

All the analysis was performed in python using the scipy, numpy and pandas packages. We estimated odds ratios for top vs. bottom income groups via survey-weighted logistic regression, adjusted for urban/rural location of dwelling, sex, education, and age (continuous). Reference categories were the bottom income quintile, rural residence, female sex, and less than 9 years of completed education. In order to deal only with binary variables, the education variable is divided in two classes depending on whether the individual has completed or not 9 years of school, and self-rated health is divided into “poor/fair” vs. the remaining categories, as described in Section 2.2. We dichotomized these two inherently ordinal variables to ensure a threshold that is directly comparable across the two survey instruments, whose original response scales are not identically coded, and to keep the resulting odds ratios interpretable in the same top-vs.-bottom-quintile framework used throughout the analysis; we acknowledge that this involves some loss of information relative to the full ordinal scale, particularly for self-rated health, and return to this point in the Discussion. To properly evaluate statistical significance and reflect the complex sample design in PNS data, we used cluster-robust standard errors with primary sampling unit (PSU) and within-stratum clustering. For PSID data, where strata and PSU are not available, we used cluster-robust standard errors at the family/household level to account for within-family correlations. Statistical weights obtained from the surveys were assigned to each observation. Individuals with missing values on the outcome, exposure, sampling weight, cluster identifier, or any adjustment covariate for a given model were excluded from that model (complete-case analysis); the resulting analytic sample size and number of events for each model are reported in Supplementary Table S1. The weighted Generalized Linear Model regression with binomial response distribution was implemented in python using the statsmodel.api.GLM package (v 0.13.2 https://www.statsmodels.org (24)). In practice, the probability *p* for an event was fitted with the logistic equation


$$\begin{array}{c} \log\left(\frac{p}{1-p}\right)=\beta_{0}+\beta_{Q}X_{Q}+\beta_{U}X_{U}+\beta_{S}X_{S}+\beta_{E}X_{E}+\beta_{A}X_{A} \end{array}$$ (1)

where *X*_*Q*_, *X*_*U*_, *X*_*S*_, *X*_*E*_ and *X*_*A*_ are the predictor variables for income quintile (top or bottom), urban/rural location of dwelling, sex, education, and age (continuous), respectively, and β_0_, β_*Q*_, β_*U*_, β_*S*_, β_*E*_ and β_*A*_ are the model coefficients that were obtained solving the maximum likelihood score equations, using iteratively reweighted least squares. Age was added in Equation 1 as a covariate to control for residual within-quintile age differences inside each age stratum defined in Section 2.2; as a complementary descriptive check, we also verified the weighted mean age in the bottom and top income quintiles within each stratum. This age-adjusted specification also served as a sensitivity analysis relative to our earlier, unadjusted specification, and did not alter the qualitative pattern of results reported below except where explicitly noted.

To directly test whether the income-health gradient differs between Brazil and the USA, rather than relying on visual comparison of the two countries' separately estimated confidence intervals, we additionally fitted, for each outcome, a single pooled logistic model in which country was interacted with income and with every other covariate (urban/rural location, sex, education, and age). This fully-interacted specification is mathematically equivalent to fitting the two country-specific models separately, reproducing identical country-specific odds ratios, while additionally yielding a valid joint Wald test of the income × country interaction term, which we report alongside the country-specific results in Section 3.2.

Linear cross-correlation analysis was performed by calculating the Pearson coefficients, using the scipy.stats.pearsonr python package, between the state-level prevalence of each health condition and the state-level percentage of the population in the bottom income quintile, separately for each country. This ecological (aggregate-level) analysis was intended to characterize the geographic distribution of the income-health relationship within each country, complementing the individual-level regression analysis described above; as such, and as we note in the Results and Discussion, its results cannot be interpreted as evidence of an individual-level association.

## 3 Results

### 3.1 A comparison of health indicators in the USA and Brazil

A standard health indicator like the life expectancy at birth has been generally increasing both in the USA and in Brazil in the past 60 years (Figure 1a). Life expectancy in Brazil was relatively low in 1960 (53 years) and exceeded 72 years in 2022, a value that is getting close to the one reported for the USA (76 years). The gap between the two countries is closing also with respect to infant and child mortality (Figures 1b, c, respectively). Brazil in 1960 had extremely high mortality rates (12.5 and 5%, for infant and child mortality, respectively) which are now reduced to 1.2 and 0.15%, approaching the values reported for the USA (0.5 and 0.09%).

When we look at more specific health indicators obtained from the surveys, we notice that the health conditions in the two countries are generally similar, or even slightly better in Brazil than in the USA (Figure 1). In particular, a much larger fraction of the population, irrespective of age, declares to be in poor or fair health conditions in the USA as in Brazil (Figure 1d). The prevalence of hypertension (Figure 1e) and diabetes (Figure 1f) are similar in the two countries for all age groups. Obesity is more prevalent in the USA than in Brazil, especially among the young (18–25 years), where the prevalence in the USA is more than twice as large than in Brazil (Figure 1g). Cancer diagnoses are in general more prevalent in the USA than in Brazil, especially for people aged 65 or more (Figure 1h). This might reflect both a slightly older population and a wider availability of cancer screening and testing in the USA with respect to Brazil. Finally, psychiatric conditions are more prevalent in the USA with respect to Brazil over all age groups, but especially among the younger population. In the USA 15% of the individuals in 18–25 years bracket is affected by mental health issues, as compared with around 7% in Brazil (Figure 1i).

### 3.2 Income inequality and health

Income and wealth inequalities are larger in Brazil than in the USA, but how does this translate into health disparities among different income classes? To answer this question, we subdivided the individuals considered in the surveys into income quintile classes, as discussed in the section 2.3. We then studied how the health indicators reported in Figure 1 depended on the income class in the two countries. We also considered possible confounding factors such as sex, location of housing (urban or rural), education (whether the individual had completed 9 years of schooling), and age. All these factors were reported in both surveys. When performing the comparison, we estimated odds ratios (OR) and confidence intervals (CI) through a multiple logistic regression, and tested the country × income interaction as described in section 2.4. Table 1 summarizes the resulting income-quintile odds ratios for both countries alongside the corresponding interaction *p*-value for each of the six outcomes; the full covariate detail underlying these estimates is reported in Supplementary Tables S2, S3.

**Table 1:** Income-quintile odds ratios (top vs. bottom quintile) and country × income interaction test, by condition.

Condition	Brazil OR (95% CI)	USA OR (95% CI)	Interaction *p*
Self-rated health (18–50)	0.19 (0.11, 0.31)	0.16 (0.10, 0.24)	0.606
Hypertension (≥60)	0.88 (0.69, 1.13)	0.81 (0.57, 1.15)	0.699
Diabetes (≥60)	0.92 (0.66, 1.29)	0.49 (0.31, 0.78)	0.029
Obesity	0.93 (0.63, 1.37)	0.53 (0.44, 0.63)	0.010
Cancer diagnosis (≥60)	1.72 (0.97, 3.06)	1.21 (0.77, 1.88)	0.339
Psychiatric condition	1.77 (1.43, 2.19)	0.47 (0.36, 0.61)	< 10^−14^

Odds ratios are adjusted for urban/rural residence, sex, education, and age; the interaction *p*-value tests whether the income log-odds-ratio differs between the two countries (see Section 2.4). Obesity odds ratios use measured anthropometric data for Brazil and self-reported data for the USA (see main text and Supplementary Table S2 for the Brazilian self-reported sensitivity analysis).

We note for interpretation that the age odds ratios reported in Supplementary Tables S2, S3 are, by construction, close to 1 for every outcome: unlike income, sex, education, and urban/rural residence, which compare two discrete groups, age enters the model as a continuous covariate, so its odds ratio represents the multiplicative change in odds per single additional year rather than a group-level contrast. A per-year OR of, for example, 1.06 (as observed for self-rated health in Brazil) compounds substantially over the 18–50 age range studied for that outcome (approximately 1.06^30^≈5.7-fold), and should not be compared directly, in raw magnitude, to the income, sex, education, or urban/rural odds ratios reported alongside it. Because the true per-year effect size differs across the six outcomes, and because the Brazilian sample is roughly 20 times larger than the USA sample, the same underlying per-year effect is statistically detectable in one country but not the other for some conditions (e.g., diabetes, where the age odds ratio is significant in Brazil but not in the USA); this may reflect the difference in statistical power between the two surveys rather than a difference in the underlying age relationship.

We first considered self-reported health conditions, concentrating on adults aged between 18 and 50, thus minimizing the effects of the chronic conditions that are more prevalent for older individuals. Figure 2a shows that individuals belonging to the bottom 20% of the income distribution generally claim to have poorer health than those belonging to the upper 20% (see Supplementary Tables S1–S3). In Brazil, the number of individuals with poor health is five times smaller in the top income quintile than in the bottom one [0.8%, (0.5–1.2)% 95% CI vs. 5.5%, (4.9–6.1) 95% CI, with OR = 0.19, (0.11–0.31) 95% CI, *p* < 10^−11^ after controlling for sex, rural/urban location, education, and age], and in the USA the pattern is similar [4.9%, (3.2–6.6)% 95% CI vs. 25.4%, (22.8–28)% 95% CI, with OR = 0.16, (0.10–0.24) 95% CI, *p* < 10^−18^]. The country × income interaction test shows that this relative gradient does not differ significantly between the two countries (*p* = 0.606; Table 1), even though the absolute prevalence of poor/fair health in the bottom income quintile is much larger in the USA than in Brazil: both countries show a comparable proportional reduction in the odds of poor health moving from the bottom to the top quintile, but the USA's substantially higher baseline prevalence at every income level (a country-level difference, not an income-specific one) produces a much larger absolute gap between its own top and bottom quintiles.

**Figure 2:**
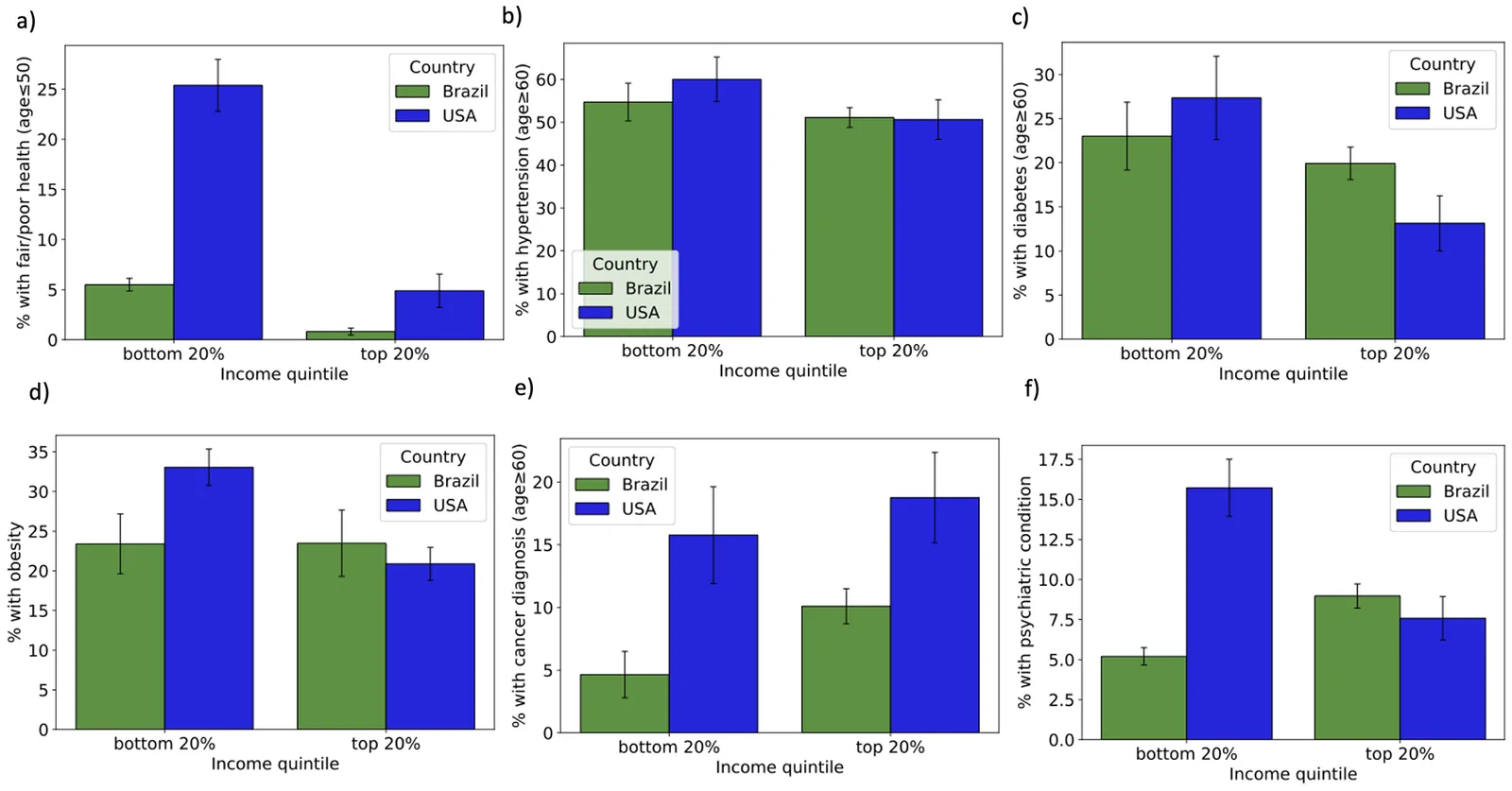
Income dependence of health status for USA and Brazil. Percentage of individuals in the top and bottom 20% of the income distribution with **(a)** fair/poor health, **(b)** with hypertension, **(c)** obese, **(d)** with diabetes, **(e)** with a cancer diagnosis, **(f)** with a psychiatric condition in the two countries. Panel **(a)** is restricted to individuals aged 18–50; panel **(c)** (obesity, measured data for Brazil and self-reported data for the USA) carries no age restriction; panels **(b, d, e)** consider only individuals aged 60 or more. Error bars represent 95% confidence intervals.

We next considered specific conditions and risk factors (see Figure 2 and Supplementary Tables S1–S3). Hypertension in individuals aged 60 or more displays no significant income dependence in Brazil [51.1% (48.8–53.4)% 95% CI for top earners and 54.7%, (50.3–59.1)% 95% CI for bottom ones, with OR = 0.88, (0.69–1.13) 95% CI, not significant] while in the USA top earners have lower prevalence with respect to bottom ones although not significant after correcting for education, sex, location, and age [50.6%, (46–55.2)% 95% CI for top earners and 60%, (54.8–65.2)% 95% CI for bottom ones, with OR = 0.81, (0.57–1.15) 95% CI, not significant]; the country × income interaction is not significant either (*p* = 0.699), so the data do not support a cross-country difference in the income-hypertension gradient. Diabetes in individuals aged 60 or more again displays no significant income dependence in Brazil [19.9%, (18.1–21.8)% 95% CI for top earners vs. 23%, (19.2–26.8)% 95% CI, for bottom ones, with OR = 0.92, (0.66–1.29) 95% CI, not significant] while in the USA there is a significant difference in the prevalence between top and bottom earners that remains significant even after adjustments [13.1%, (10–16.2)% 95% CI for top earners vs. 27.3%, (22.6–32.1)% 95% CI for bottom ones, with OR = 0.49, (0.31–0.78) 95% CI, *p* = 0.002]; in this case the country × income interaction is also significant (*p* = 0.029), formally confirming that the diabetes income gradient is steeper in the USA than in Brazil.

In the case of obesity, the two countries differed markedly in their association with income (Figure 2d). Notice that the Brazilian survey includes information on self-reported and measured anthropometric data used to define obesity, but both types of data show the same trend, as shown in Supplementary Figure S1; we prioritize the measured data for Brazil as our principal analysis because measured height and weight are generally more valid than self-reported values (25). Using measured data, we observed a similar prevalence for bottom (23.4%, [19.6–27.2]% 95% CI) and top income earners in Brazil [23.5%, (19.3–27.7)% 95% CI], with OR = 0.93, (0.63–1.37) 95% CI, not significant. As a sensitivity analysis, we repeated this comparison using the larger self-reported BMI sample available for Brazil; after the same adjustment, this analysis likewise showed no significant income association [OR = 1.01, 95% CI (0.89, 1.15), *p* = 0.87], consistent with the measured-BMI result. In the USA, where only self-reported anthropometric data are available, the prevalence of obesity is much smaller among individuals in the top 20% of the income distribution [20.9%, (18.8–23)% 95% CI] than in those in the bottom 20% [33.1%, (30.8–35.3)% 95% CI], with OR = 0.53, (0.44–0.63) 95% CI, *p* < 10^−12^. The country × income interaction is significant (*p* = 0.010), formally confirming a steeper obesity income gradient in the USA than in Brazil. Given the number of comparisons performed across the six outcomes examined in this study, this and the other individual results should nonetheless be interpreted with appropriate caution.

The rate of cancer diagnosis displays no statistically significant dependence on income in the USA [OR = 1.21, (0.77–1.88) 95% CI, not significant]. In Brazil, people in the bottom 20% of the income distribution reported fewer cancer diagnoses [4.6%, (2.8–6.5)% 95% CI] than those in the top 20% [10.1%, (8.7–11.5)% 95% CI]; however, after adjusting for age this association is no longer statistically significant [OR = 1.72, (0.97–3.06) 95% CI, *p* = 0.064] (Figure 2e), consistent with the non-significant country × income interaction for this outcome (*p* = 0.339). Finally, psychiatric conditions depended on income both in Brazil and the USA, but the dependence was inverted. In Brazil, top earners were more affected [9.0%, (8.2–9.7)% 95% CI] than low earners [5.2%, (4.7–5.7)% 95% CI], with OR = 1.77, (1.43–2.19) 95% CI, *p* < 10^−7^, while in the USA high income individuals suffered from mental health issues at much lower rates [7.6%, (6.2–8.9)% 95% CI] than low-income ones [15.7%, (13.9–17.5)% 95% CI], with OR = 0.47, (0.36–0.61) 95% CI, *p* < 10^−8^, after adjustments (Figure 2f). The country × income interaction for psychiatric conditions is highly significant (*p* < 10^−14^); we emphasize that this reflects a genuine reversal in the direction of the income association, rather than simply a larger gradient of the same sign, and interpret it accordingly in the Discussion.

### 3.3 Geographical aspects of income and health inequality

To illustrate how income and health disparities are geographically distributed in the USA and in Brazil, we associate to each household in the surveys an income quintile based on the income distribution of the entire country. We then compute the fraction of households in each state that belong to the bottom and top quintiles, which we denote as *f*_*b*_ and *f*_*t*_, respectively. Data on Brazil display a clear north-south divide, where northern states have higher *f*_*b*_ and lower *f*_*t*_ than southern states (Figures 3a, d). In the USA, the income divide is less homogeneous apart from midwest and southern states, where the concentration of low income households is more prevalent (Figures 3b, e). To summarize this state-by-state variation in a single view, Figures 3c, f show the distribution (histogram) of *f*_*b*_ and *f*_*t*_ values across all states, separately for each country. This distribution confirms that Brazil is very polarized, with states clustering into two distinct groups with respect to income—broadly, a low-*f*_*b*_/high-*f*_*t*_ southern cluster and a high-*f*_*b*_/low-*f*_*t*_ northern cluster—whereas the corresponding USA distribution is unimodal and comparatively narrow, indicating a less polarized geographical distribution of income classes across states (Figures 3c, f).

**Figure 3:**
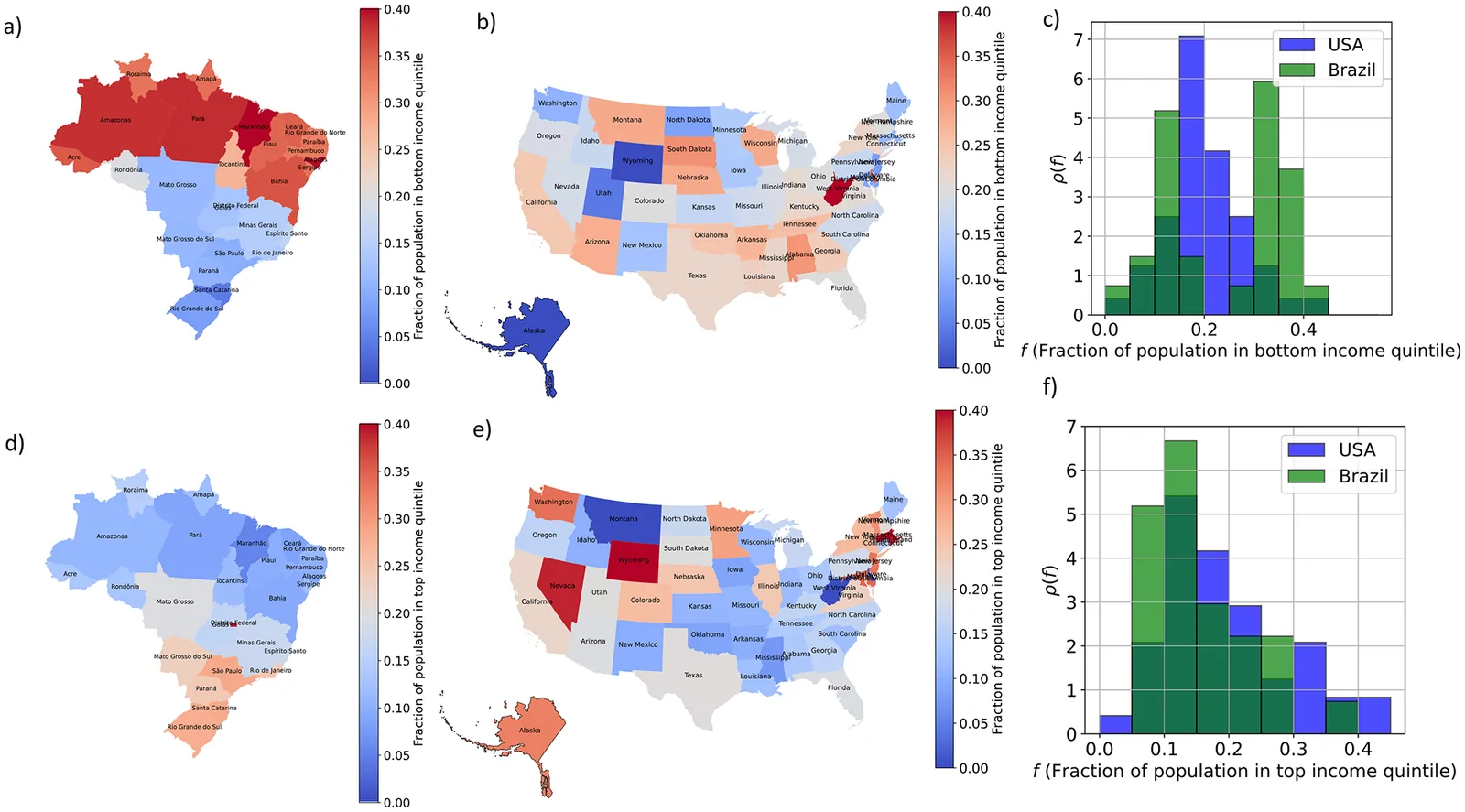
Geographic dependence of income in Brazil and USA. Fraction of households *f*_*b*_ in the bottom 20% of the national income distribution in each state of **(a)** Brazil and **(b)** USA. **(c)** Distribution of *f*_*b*_ in the two countries. Fraction of households *f*_*t*_ in the top 20% of the national income distribution in each state of **(d)** Brazil and **(e)** the USA. **(f)** Distribution of *f*_*t*_ in the two countries.

We next considered how health indicators are distributed geographically within the two countries, in relation to the income level of each state. As this analysis correlates state-level (aggregate) health prevalence with the state-level (aggregate) percentage of the population in the bottom income quintile, it is an ecological analysis; the resulting correlations describe the geographic co-distribution of income and health at the state level and cannot be interpreted as evidence of an individual-level association between income and health, which is instead addressed directly by the regression analysis in Section 3.2. To this end, we computed the percentage of population reporting fair/poor health as well as the prevalence of hypertension, diabetes, obesity, psychiatric conditions and cancer for each state in Brazil and USA. We then associated these conditions with the percentage of household belonging to the bottom income quintile in each state for the two countries (see Figure 4 and Supplementary Table S4). As shown in Figure 4a, the percentage of population declaring fair/poor health was significantly correlated with the percentage of the population in the bottom income quintile, both in the USA (*r* = 0.34, *p* = 0.02) and Brazil (*r* = 0.73, *p* < 10^−5^). The prevalence of hypertension in each state (Figure 4b) was not significantly correlated with the percentage of the population in the bottom income quintile for both countries (*p*>0.05). Repeating the same analysis for the other conditions in the USA, we found no correlation for diabetes (*p*>0.05), obesity and psychiatric conditions, and anti-correlation for cancer implying that poorer states displayed lower prevalence (*r* = −0.31, *p* = 0.03, Figure 4f). For Brazil, we found no correlation for diabetes and obesity (*p*>0.05), while we observed statistically significant anti-correlation for psychiatric conditions (*r* = −0.7, *p* = 10^−5^) and cancer (*r* = −0.80, *p* < 10^−7^). We note that Brazil and the USA are represented by different numbers of geographic units (27 states/federal units vs. 50 states, respectively), which affects the precision and comparability of these correlation coefficients between the two countries; we return to this point as a limitation in the Discussion.

**Figure 4:**
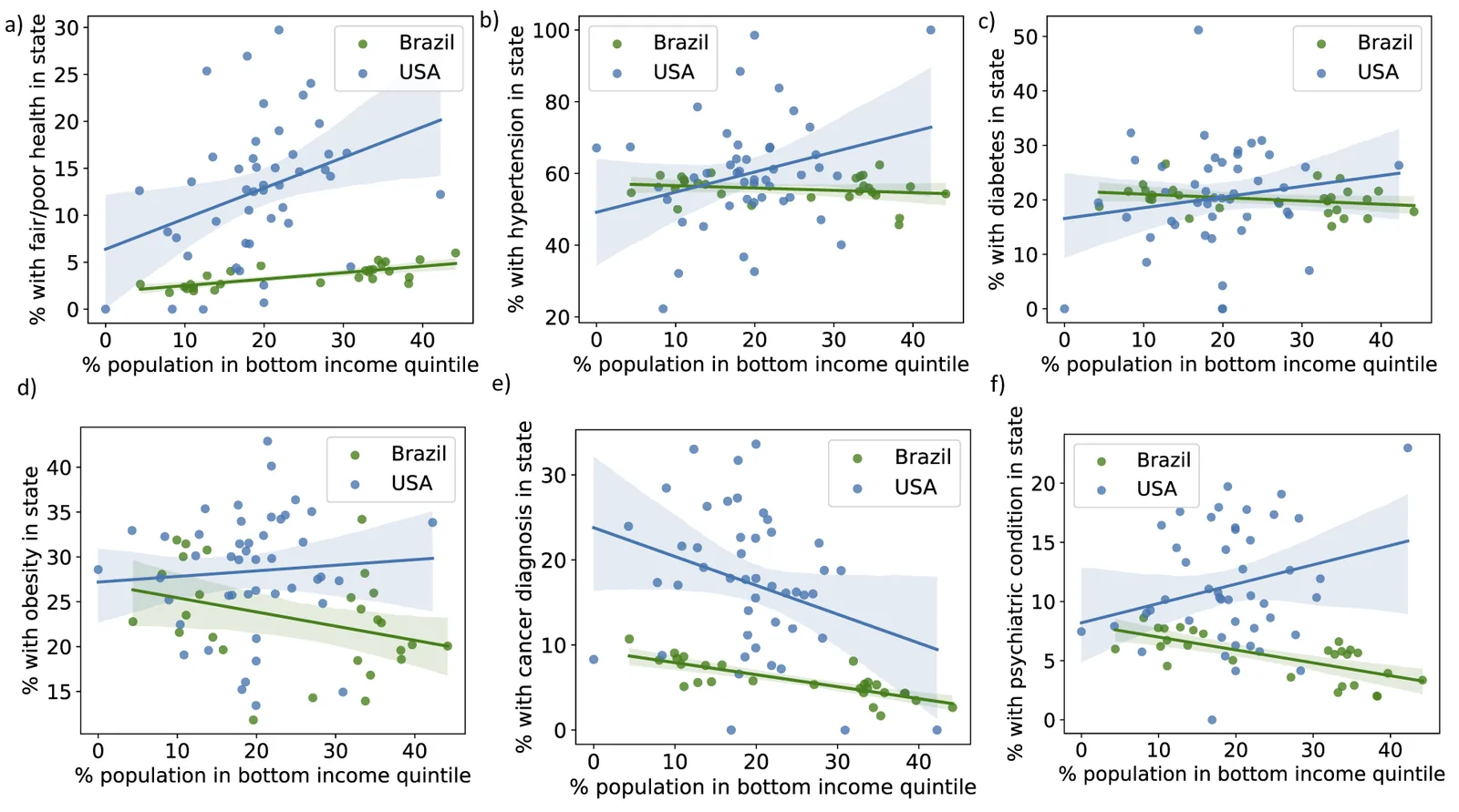
Dependence of health conditions on low income population in the USA and Brazilian states. Percentage of individuals with **(a)** fair/poor health, **(b)** with hypertension, **(c)** obese, **(d)** with diabetes, **(e)** with a psychiatric condition and **(f)** with a cancer diagnosis as a function of the percentage of individuals in the bottom quintile of the income distribution in each state. The line is a linear regression fit and the shaded area represents the 95% confidence interval. Panel **(a)** is restricted to individuals aged 18–50; panels **(c, e)** carry no age restriction; panels **(b, d, f)** consider only individuals aged 60 or more.

It is interesting to compare in more detail two cases where the results in the two countries diverge: obesity and psychiatric conditions. In Brazil, the prevalence of obesity was uniformly distributed across the states, with a rich state like São Paulo displaying a slightly higher rate (Figure 5a). Conversely, in the USA, the geographical distribution of obesity was more heterogeneously distributed (Figure 5b). We investigated if the relations were related to differences in living conditions of the population in each state in terms of urban and rural locations. Looking at the comparison between households living in rural and urban areas, we found that in Brazil obesity was larger in urban areas [OR = 1.47, (1.31–1.65) 95% CI, *p* < 10^−10^, according to self-reported data which have much larger sample size than measured ones], while in the USA, we observed only a small difference [OR = 0.79, (0.64–0.98) 95% CI, *p* = 0.034] in obesity between urban and rural areas (Figure 5c and Supplementary Table S2). When looking at psychiatric conditions, we found that in Brazil there was a slightly larger prevalence in the southern and richer states (Figure 5d), while in the USA, we observed again a close similarity with the geographical distribution of income. Psychiatric problems are again more prevalent in midwestern and southern states than in the rest of the country (Figure 5e). Finally, psychiatric conditions are more prevalent in urban areas in Brazil [OR = 1.56, (1.24–1.96) 95% CI, *p* < 10^−4^], while they are similar in urban and rural areas in the USA (Figure 5f and Supplementary Table S2).

**Figure 5:**
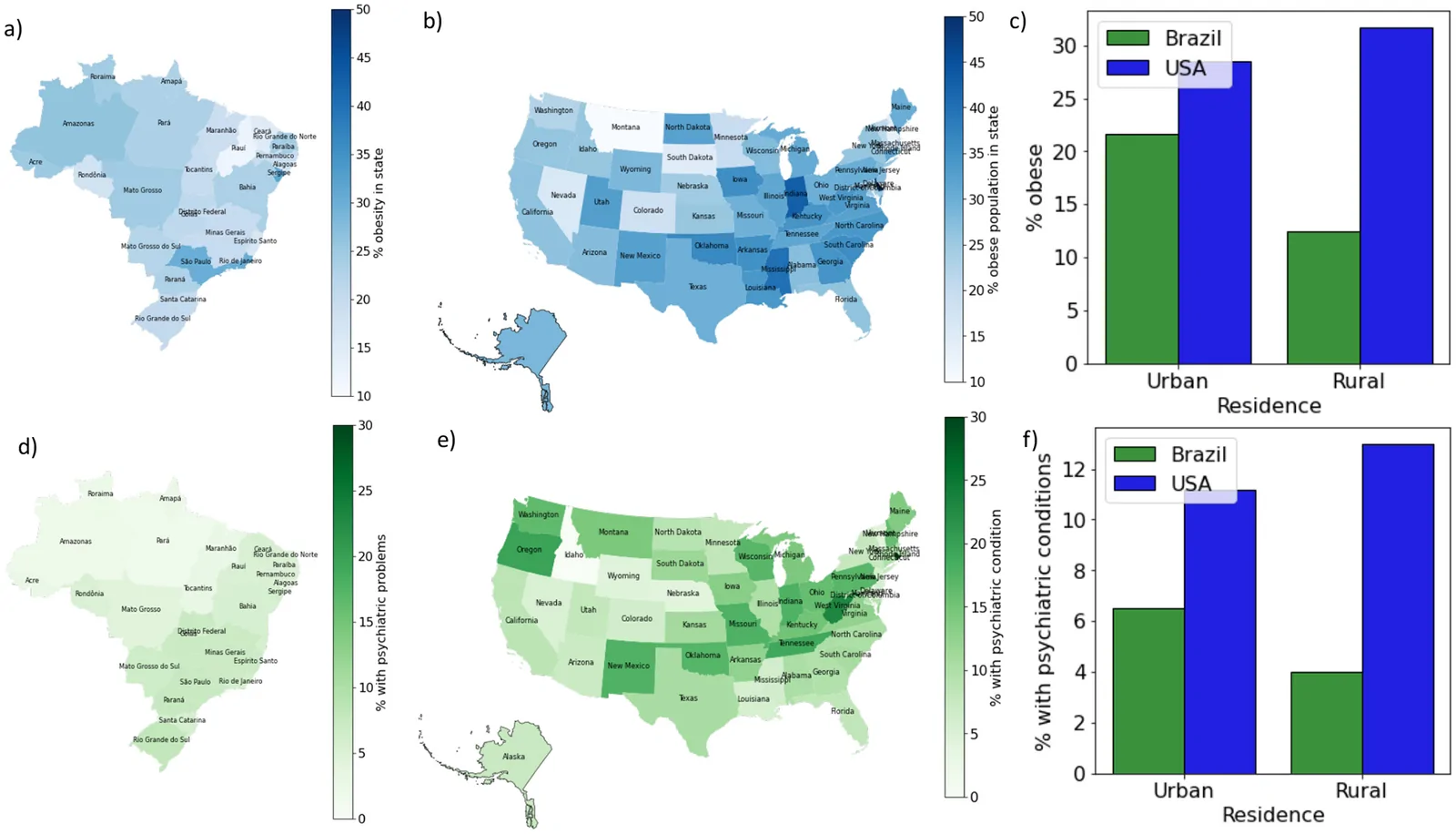
Geographic dependence of obesity and psychiatric conditions in Brazil and USA. Prevalence of obesity (self-reported data) in different states in **(a)** Brazil and **(b)** USA. **(c)** Prevalence of obesity (self-reported data) in rural and urban areas for the two countries. Percentage of individuals with psychiatric conditions in the different states of **(d)** Brazil and **(e)** USA. **(f)** Percentage of individuals with psychiatric conditions in rural and urban areas for the two countries.

### 3.4 Reliance on healthcare across income groups

The healthcare system is different in the USA and Brazil. In contrast to the USA, where healthcare access largely depends on employment and private insurance, Brazil guarantees universal healthcare coverage to all citizens through its public health system, the Sistema Único de Saúde (SUS), established under the 1988 Brazilian Constitution (Article 196), which ensures that healthcare is a right for all Brazilians and a duty of the state (18). We quantified the reliance on healthcare for different income groups in the two countries using survey data for the year 2019. In the USA, a large part of the population (59%) relies on private health insurance (Supplementary Tables S5, S6), but public coverage is available in some cases, such as Medicare for people older than 65 and Medicaid for low-income people. As shown in Figure 6a, in the USA 86.5% of the individuals in the top 20% income group rely on private insurance, compared with only 25.3% in the bottom 20% group. Conversely, only 3.1% of the individual in the top income group are uninsured, compared with 18.5% in the bottom income group.

**Figure 6:**
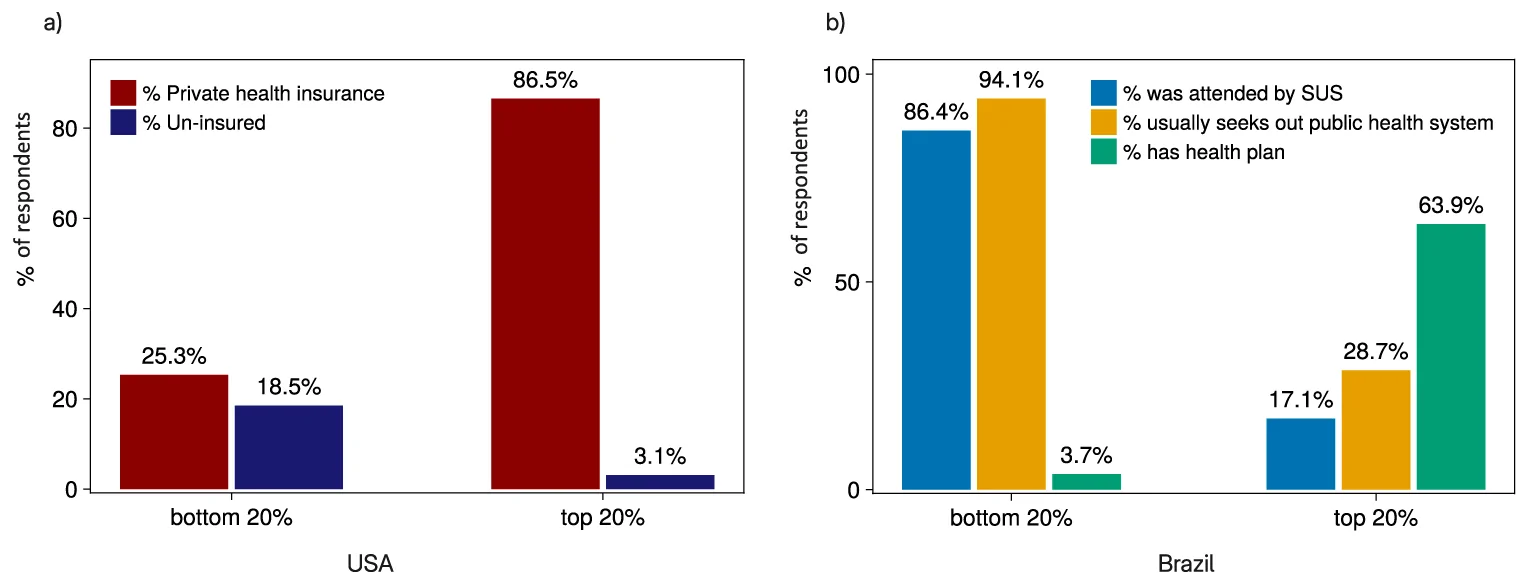
Reliance on health insurance for different income groups in the USA and Brazil. **(a)** Percentage of respondents with private health insurance or uninsured by family income quintile in the 2019 National Health Interview Survey (NHIS) for the USA. Percentages are weighted to reflect the U.S. population. **(b)** Income-based reliance on public and private health systems in Brazil, according to the 2019 National Health Survey (Pesquisa Nacional de Saúde, PNS). The figure illustrates the percentage of individuals from the bottom and top 20% income groups who were attended by the SUS (Unified Health System), usually seek public health services, or have private health insurance.

In Brazil, we saw that a large part of the bottom 20% income group relied (94.1%) or was attended (86.4%) by SUS, while 3.7% had a private insurance (Figure 6b). A large fraction of the individuals in the top 20% income group had a private health insurance (63.9%), but many relied on SUS (28.7%). We refined the analysis considering the reliance on SUS for different regions in Brazil (See Supplementary Tables S7–S9). The pattern is consistent across all regions, though its magnitude varies. In the Southeast, SUS use falls sharply from 82.6% in the bottom quintile to only 12.3% in the top, reflecting the greater concentration of high-income households in this region. By contrast, in the North and Northeast, reliance on SUS remains higher across all income groups, consistent with the regional distribution of wealth, given that these regions include some of the poorest states in the country. The results underscore the dependence on income of the access to healthcare in the two countries. While in Brazil lower-income groups rely heavily on SUS, in the USA they face the dual risk of limited private coverage and higher probability of being uninsured.

Finally, we studied how out-of-pocket (OOP) health expenditure depends on income in the two countries. In the USA, households in the bottom income quintile spend nearly half of their income (48.2%) on OOP health expenses, while those in the top quintile spend only 5%. The pattern is consistent across regions, with the Northeast showing the most extreme burden (68.8% among the poorest). Although absolute OOP spending is greater for wealthier families, their much higher incomes mean that the relative burden is minimal. This is in contrast to Brazil, where households in the top and bottom income groups devote roughly 10%–12% of their income to medicines and consultations. Regional patterns in Brazil are more heterogeneous, and we report them here descriptively, without formal statistical testing. In the South, the bottom quintile spends 14.0% of income on out-of-pocket health care vs. 9.3% for the top quintile. In the Southeast, the proportions are 13.7% for the bottom quintile and 13.1% for the top quintile. In the North, the figures are 8.0 vs. 5.6%, and in the Midwest, 14.1 vs. 11.0%. In the Northeast, the bottom quintile spends 11.7 vs. 12.7% for the top quintile. In the USA, by contrast, the healthcare system places a disproportionate financial strain on the poor in every region, reflecting the absence of universal protection.

## 4 Discussion

According to the fundamental cause theory, proposed by Link and Phelan (19), SES affects multiple disease conditions through multiple mechanisms, such as lifestyle or diet, maintaining an association with health even after a change of the specific disease risk factor. For this reason, it is imperative to characterize the impact of SES on health studying a variety of diseases and health outcomes. This is the approach taken in the present paper, where we analyzed how the prevalence of obesity, diabetes, hypertension, cancer, psychiatric conditions and self-reported health varied across income groups in Brazil and the USA.

Brazil and the USA are two large federal countries, covering a very large area and composed by a very diverse population. Both countries are highly unequal in terms of income and wealth distribution: Brazil has been consistently unequal in the past decades, while in the USA inequality has been on the rise (9). The World Bank's Human Capital Index (see https://databank.worldbank.org/source/human-capital-index) estimates that a child born in Brazil will achieve approximately 55% of the productivity expected under conditions of complete education and full health. This indicator highlights continuing limitations in access to quality health and education, although it is not itself a direct measure of income inequality. A high inequality usually also involves disparities in the access to healthcare services and resources, which is often limited in rural areas and regions with a high poverty rate (26).

The foundational differences in healthcare systems in the two countries shapes the ways in which health inequalities manifest themselves, with Brazil's system providing a framework for more equitable access despite regional and socioeconomic disparities (18). According to a recent editorial, Brazil is trying to balance unequal access to healthcare, especially in rural and impoverished areas, through investment in infrastructure, training, and technology like telemedicine (27). Moreover, Brazil needs to improve the quality of healthcare by reducing wait times, enhancing medical professional training, and ensuring adequate equipment and resources, improving coordination between different care levels by implementing integrated electronic medical records and other digital solutions (27). Overall, there are still many flaws in public Brazilian healthcare but the Brazilian national health service has provided solutions for problems as unequal access to healthcare services, low quality of care, and a lack of coordination between different levels of care. On the other hand, the USA is one of the richest countries in the world, but as discussed in recent papers, economic inequality has been increasing for decades and is now among the highest in developed countries (15). This inequality has an impact on healthcare, since 27 million Americans remain uninsured, most of whom have incomes near or below the poverty line (17).

Our analysis clearly shows that Brazil dramatically improved life expectancy at birth, reduced child mortality rate as well as infant mortality rate, approaching the levels of the USA. Our data show that in the USA the percentage of people with self-reported poor health is much larger than in Brazil, for all age groups. When analyzing the differences in specific pathologies, it is worth highlighting that both countries have a high percentage of people with hypertension, diabetes, and obesity, that has been associated in the literature with excessive consumption of ultra-processed food (28). On the other hand, the USA shows a higher percentage of cancer diagnosis, but this might also be due to delays in diagnosis in Brazil (29). The higher percentage of people with psychiatric condition in the USA with respect to Brazil describes a society with important problems related to wealth and general inequality. A recent meta-analysis showed that overweight and obesity are more prevalent in people with severe mental illness (30).

Our comparative analysis of income-related differences in health conditions, formally tested via a country × income interaction and adjusted for education, sex, rural/urban location, and age, shows that in the USA individuals in the bottom income group have significantly higher prevalence of diabetes, obesity, psychiatric condition and fair/poor self-reported health than individuals in the top income group, and that this gradient is significantly steeper than in Brazil for diabetes and obesity specifically (Table 1). In Brazil, on the other hand, only self-reported fair/poor health is larger for the low-income group than for the high-income group, while all the other conditions show no significant differences in prevalence between income groups, with the exception of psychiatric conditions, which affect more high-income individuals than low-income ones.

A plausible interpretation of the steeper USA gradient for diabetes and obesity is that these are chronic conditions whose management (regular clinical monitoring, medication adherence, access to nutritionally adequate food) depends heavily on sustained healthcare access and on the affordability of a healthy diet – both of which are more directly income-dependent in a system where insurance coverage and its quality are closely tied to employment and ability to pay, as in the USA, than in a system with constitutionally guaranteed universal coverage, as in Brazil (17, 18). An odds ratio near 1, as we observe for these two conditions in Brazil, indicates that the odds of the condition are essentially unchanged between the top and bottom income quintiles once other covariates are accounted for, consistent with a healthcare system in which access to chronic-disease management does not vary sharply by income; the odds ratios of 0.49 (diabetes) and 0.53 (obesity) we observe in the USA indicate that top-quintile individuals have roughly half the odds of these conditions relative to bottom-quintile individuals, a pattern more consistent with income-stratified access to preventive and ongoing care.

The reversal we observe for psychiatric conditions is a qualitatively different pattern and warrants separate interpretation. Rather than a difference in the magnitude of a gradient of consistent sign, higher income in Brazil is associated with a *higher* probability of a psychiatric diagnosis, while in the USA it is associated with a *lower* probability, and the country × income interaction confirms this reversal is highly significant (*p* < 10^−14^). One possible hypothesis, though not directly testable with the present data, is differential access to psychiatric diagnosis itself: if lower-income individuals in Brazil are systematically less likely to receive a formal psychiatric diagnosis even when experiencing comparable symptom burden—for instance because specialized mental-health services remain less available in lower-income areas despite SUS's nominal universality – then the observed income gradient in diagnosed prevalence would partly reflect a gradient in diagnostic access rather than in underlying prevalence. These findings may have policy implications for strengthening the availability and accessibility of mental health services in lower-income areas of Brazil in order to reduce disparities in diagnostic access and improve the early identification and treatment of mental health conditions across socioeconomic groups. In the USA, by contrast, where psychiatric care access is itself income- and insurance-dependent but where the disease burden of psychiatric conditions has also been linked in the literature to socioeconomic stress and inequality more broadly (30), the observed pattern is consistent with genuine excess prevalence among lower-income individuals. Distinguishing a diagnostic-access explanation from a genuine prevalence difference is not possible with the present cross-sectional survey data, and we flag this as an important direction for future work using clinical or symptom-based (rather than diagnosis-based) measures.

When considering additional factors besides income, we found that in Brazil higher education has a significant effect in reducing the rate of hypertension, diabetes and fair/poor health and in increasing the rate of psychiatric conditions. In the USA, higher education is significantly associated with lower prevalence of diabetes and fair/poor health and higher prevalence of psychiatric conditions, although the dominant factor remains income. When we consider the location of the household, individuals living in urban locations in Brazil have significantly higher prevalence of diabetes and psychiatric conditions, while in the USA only the rate of obesity is slightly larger for individuals living in urban areas. Differences in healthcare-system design and the broader socioeconomic and lifestyle context of the two countries offer a plausible explanation for these patterns. While lifestyle factors diet, physical activity, and related behaviors) have been identified as important determinants of these outcomes in the literature (31), they were, however, not directly examined in the present study and therefore cannot be empirically verified using the current data.

Beyond their scientific interest, these findings have direct relevance for health policy. The comparatively flat income gradient we observe for most chronic conditions in Brazil, despite Brazil's substantially higher income and wealth inequality, is compatible with, but does not demonstrate, the hypothesis that universal healthcare coverage may attenuate income-related differences in chronic-disease burden (32), and offers indirect empirical support for policy discussions around expanding healthcare-coverage guarantees in systems, such as that of the USA, where coverage remains contingent on income and employment. Conversely, the substantial USA-Brazil difference we find for out-of-pocket health expenditure (Section 3.4, Supplementary Tables S10, S11) suggests that financial-protection mechanisms, and not only nominal insurance coverage, are an important lever for reducing income-related health disparities (33).

Finally, our geospatial analysis of income dependence shows a clear division between north and south in Brazil, the latter with higher income. This is not a surprise since it is known that the south of Brazil with its mega cities like São Paulo and Rio de Janeiro play an important role for the country's economy. A recent review studied regional inequalities present in Brazil's public healthcare system from 2000 to 2016, using a typology of 438 health regions based on socioeconomic development and supply and complexity of health services (12). Moreover, this study highlights that while Brazil experienced significant progress in reducing poverty and inequality during this period, disparities persist, particularly in healthcare, between north and south of Brazil (12). Another study showed that at the individual level, female gender, advanced age, low education attainment, living without a partner and having hypertension, diabetes or depression were statistically associated with higher odds of poor self-reported health (SRH) conditions (10). In the USA the situation is more scattered, but it is interesting to point out that, while in Brazil obesity and psychiatric conditions are more present in the richest part of the country, the south, and in particular more in urban areas, in the USA the situation is different, with a higher percentage in geographic areas with low income and practically no differences can be detected between urban and rural areas.

Since our study relies on survey data where conditions are self-declared, it is important to assess how this affects the quality of the results. Previous work on Brazilian PNS has compared self-reported survey data with measurements for BMI (25, 34) (see also Supplementary Figure S1), hypertension (35) and diabetes (36) showing a reasonably good accuracy. Investigation on self-reported cancer diagnosis in different surveys (37–40) showed significant errors that were, however, randomly distributed across different social groups, so that there is no systematic reporting bias due to socio-economic factors (40).

Several further limitations of this study should be noted. First, and most importantly, our data are cross-sectional, and the associations we report—however robustly estimated—cannot be interpreted as evidence of a causal effect of income on health, nor of a causal effect of healthcare-system design on the size of the income-health gradient; the explanations we offer above for the observed cross-country differences are plausible hypotheses motivated by known institutional differences between the two healthcare systems, not causal claims supported by the study design. Second, we used household income rather than an asset- or wealth-based measure of socioeconomic position, because it was the socioeconomic indicator most comparably harmonized between PNS and PSID; income is more temporally variable than wealth and may therefore be a noisier indicator of an individual's longer-run socioeconomic position. Third, self-rated health and education were dichotomized for comparability across the two survey instruments and interpretability of the resulting odds ratios, at the cost of some loss of information relative to their original, more finely graded scales, particularly for the inherently ordinal self-rated health measure. Fourth, the age-restriction scheme used to define each outcome's analytic sample (Section 2.2) leaves individuals aged 51–59 uncovered by the self-rated-health and chronic-disease models specifically, though this age band remains included in the psychiatric-condition and obesity analyses, which carry no age restriction. Fifth, the state-level ecological correlations reported in Section 3.2 are based on different numbers of geographic units in the two countries (27 Brazilian states/federal units vs. 50 US states), which affects the precision and cross-country comparability of the corresponding correlation coefficients. Sixth, although self-reported and measured obesity data show consistent trends within Brazil (Supplementary Figure S1), obesity in the USA is available only from self-reported height and weight; this does not establish that self-report bias in obesity is equivalent between the two countries, and cross-country obesity comparisons should be interpreted with this residual limitation in mind. Finally, we examined six health outcomes using the same top-vs.-bottom-quintile comparison in each country; given the number of comparisons performed, individual results—particularly those closer to the conventional significance threshold—should be interpreted with appropriate caution rather than in isolation. Finally, we should acknowledge a broader limitation related to cross-country survey comparability.

All together, our comparative analysis shows that income-related health inequality is more pronounced in the USA than in Brazil for several, though not all, of the conditions examined—specifically diabetes, obesity, and psychiatric conditions, the latter showing a reversal in direction rather than simply a difference in magnitude—despite the fact that in Brazil wealth and income inequalities are larger than in the USA. This pattern may be associated with differences in the healthcare systems and lifestyles of the two countries, though, as noted above, the cross-sectional design and empirical strategy employed in this study do not support a causal interpretation of the results.

## Data Availability

Publicly available datasets were analyzed in this study. This data can be found here: https://www.ibge.gov.br/; https://psidonline.isr.umich.edu/.
