# Supplementary material for: Differences in income-related health inequalities in Brazil and the USA: a comparative cross-sectional study
Source: Front Public Health. 2026 Oct 13;14:1917651. doi: 10.3389/fpubh.2026.1917651 (PMC13729126; doi:10.3389/fpubh.2026.1917651)
Supplement: Supplementary file 1 [file Data_Sheet_1.pdf]

Supplementary Material

SUPPLEMENTARY TABLES

Condition	Country	Income quintile	n	n (yes)	Prevalence	95% CI
Cancer	Brazil	Bottom	1447	53	4.6%	[2.8–6.5]%
(Age>60)		Top	4995	508	10.1%	[8.7–11.5]%
	USA	Bottom	649	89	15.8%	[11.9–19.6]%
		Top	570	100	18.8%	[15.2–22.3]%
Diabetes	Brazil	Bottom	1355	291	23.0%	[19.2–26.8]%
(Age>60)		Top	4941	940	19.9%	[18.1–21.8]%
	USA	Bottom	649	204	27.3%	[22.6–32.1]%
		Top	569	72	13.1%	[10.0–16.2]%
Fair/poor health	Brazil	Bottom	15756	921	5.5%	[4.9–6.1]%
(Age<50)		Top	7739	70	0.8%	[0.5–1.2]%
	USA	Bottom	2502	553	25.4%	[22.8–28.0]%
		Top	1063	57	4.9%	[3.2–6.6]%
Hypertension	Brazil	Bottom	1423	760	54.7%	[50.3–59.1]%
(Age>60)		Top	4975	2615	51.1%	[48.8–53.4]%
	USA	Bottom	648	421	60.0%	[54.8–65.2]%
		Top	570	290	50.6%	[46.0–55.2]%
Obesity	Brazil	Bottom	1565	376	23.4%	[19.6–27.2]%
(measured)		Top	1038	248	23.5%	[19.3–27.7]%
Obesity	Brazil	Bottom	20583	4003	19.7%	[18.7–20.6]%
(self-reported)		Top	20583	3328	20.7%	[19.7–21.8]%
	USA	Bottom	3694	1420	33.1%	[30.8–35.3]%
(self-reported)		Top	2165	491	20.9%	[18.8–23.0]%
Psychiatric	Brazil	Bottom	20583	883	5.2%	[4.7–5.7]%
		Top	16483	1254	9.0%	[8.2–9.7]%
	USA	Bottom	3668	498	15.7%	[13.9–17.5]%
		Top	2155	168	7.6%	[6.2–8.9]%

Table S1. Crude prevalence for hypertension, diabetes, cancer, obesity, fair/poor health and psychiatric conditions for individuals in the two surveys, subdivided according to their income quintile (top and bottom 20%).

Condition	Country	Variable	OR	95% CI	p-value
Hypertension	Brazil	Income (top vs bottom)	0.88	[0.69, 1.13]	0.310
		Urban vs rural	1.21	[0.93, 1.57]	0.148
		Male vs Female	0.72	[0.60, 0.85]	$< 10^{-4}$
		Education	0.76	[0.63, 0.91]	0.004
		Age (years)	1.02	[1.01, 1.04]	$< 10^{-5}$
Hypertension	USA	Income (top vs bottom)	0.81	[0.57, 1.15]	0.237
		Urban vs rural	1.00	[0.66, 1.51]	0.984
		Male vs female	1.19	[0.89, 1.58]	0.239
		Education	0.61	[0.37, 1.01]	0.056
		Age (years)	1.02	[1.00, 1.04]	0.040
Diabetes	Brazil	Income (top vs bottom)	0.92	[0.66, 1.29]	0.637
		Urban vs rural	1.59	[1.11, 2.27]	0.011
		Male vs Female	1.33	[1.06, 1.67]	0.013
		Education	0.67	[0.52, 0.88]	0.004
		Age (years)	1.01	[1.00, 1.03]	0.047
Diabetes	USA	Income (top vs bottom)	0.49	[0.31, 0.78]	0.002
		Urban vs rural	0.84	[0.51, 1.37]	0.474
		Male vs female	1.09	[0.75, 1.58]	0.644
		Education	0.47	[0.28, 0.80]	0.005
		Age (years)	0.99	[0.97, 1.02]	0.527
Obesity (measured)	Brazil	Income (top vs bottom)	0.93	[0.63, 1.37]	0.704
		Urban vs rural	1.04	[0.75, 1.46]	0.802
		Male vs Female	0.75	[0.53, 1.06]	0.099
		Education	0.94	[0.68, 1.31]	0.732
		Age (years)	1.01	[1.00, 1.02]	0.049
Obesity (self-reported)	Brazil	Income (top vs bottom)	1.01	[0.89, 1.15]	0.873
		Urban vs rural	1.47	[1.31, 1.65]	$< 10^{-10}$
		Male vs Female	0.84	[0.76, 0.93]	$< 10^{-4}$
		Education	0.84	[0.75, 0.95]	0.004
		Age (years)	1.01	[1.00, 1.01]	$< 10^{-5}$
Obesity (self-reported)	USA	Income (top vs bottom)	0.53	[0.44, 0.63]	$< 10^{-12}$
		Urban vs rural	0.79	[0.64, 0.98]	0.034
		Male vs female	1.09	[0.93, 1.27]	0.292
		Education	1.16	[0.86, 1.55]	0.333
		Age (years)	1.00	[0.99, 1.00]	0.191

Table S2. Odds ratios obtained from logistic regression using a generalized linear model with survey weights and cluster-robust standard errors, controlling for income quintile, rural/urban location, sex, educational attainment, and age (continuous). Data for Brazil were clustered by PSU within strata. Data for USA were clustered within household.

Condition	Country	Variable	OR	95% CI	p-value
Psychiatric	Brazil	Income (top vs bottom)	1.77	[1.43, 2.19]	$< 10^{-7}$
		Urban vs rural	1.56	[1.24, 1.96]	$< 10^{-4}$
		Male vs Female	0.47	[0.40, 0.56]	$< 10^{-17}$
		Education	1.19	[0.96, 1.46]	0.105
		Age (years)	0.99	[0.98, 0.99]	$< 10^{-5}$
Psychiatric	USA	Income (top vs bottom)	0.47	[0.36, 0.61]	$< 10^{-8}$
		Urban vs rural	0.79	[0.59, 1.05]	0.107
		Male vs female	0.58	[0.46, 0.74]	$< 10^{-6}$
		Education	1.52	[0.99, 2.35]	0.057
		Age (years)	0.99	[0.98, 1.00]	0.024
Cancer	Brazil	Income (top vs bottom)	1.72	[0.97, 3.06]	0.064
		Urban vs rural	1.38	[0.84, 2.27]	0.205
		Male vs Female	1.11	[0.82, 1.50]	0.508
		Education	1.25	[0.86, 1.81]	0.249
		Age (years)	1.03	[1.02, 1.05]	$< 10^{-4}$
Cancer	USA	Income (top vs bottom)	1.21	[0.77, 1.88]	0.410
		Urban vs rural	0.81	[0.50, 1.32]	0.401
		Male vs female	1.47	[1.01, 2.15]	0.047
		Education	1.49	[0.70, 3.15]	0.301
		Age (years)	1.03	[1.00, 1.05]	0.023
Fair/poor health	Brazil	Income (top vs bottom)	0.19	[0.11, 0.31]	$< 10^{-11}$
		Urban vs rural	1.24	[0.95, 1.61]	0.107
		Male vs Female	0.52	[0.40, 0.67]	$< 10^{-7}$
		Education	0.51	[0.39, 0.68]	$< 10^{-6}$
		Age (years)	1.06	[1.05, 1.08]	$< 10^{-17}$
Fair/poor health	USA	Income (top vs bottom)	0.16	[0.10, 0.24]	$< 10^{-18}$
		Urban vs rural	0.76	[0.54, 1.06]	0.110
		Male vs female	0.93	[0.71, 1.20]	0.569
		Education	0.57	[0.36, 0.89]	0.014
		Age (years)	1.05	[1.02, 1.07]	$< 10^{-5}$

Table S3. Odds ratios obtained from logistic regression using a generalized linear model with survey weights and cluster-robust standard errors, controlling for income quintile, rural/urban location, sex, educational attainment, and age (continuous). Data for Brazil were clustered by PSU within strata. Data for USA were clustered within household.

Country	Condition	r	p-value
Brazil	Hypertension	-0.21	0.294
USA	Hypertension	0.27	0.0655
Brazil	Diabetes	-0.29	0.142
USA	Diabetes	0.16	0.303
Brazil	Health	0.73	$< 10^{-5}$
USA	Health	0.34	0.0206
Brazil	Obesity	-0.34	0.084
USA	Obesity	0.07	0.641
Brazil	Psychiatric	-0.70	$< 10^{-5}$
USA	Psychiatric	0.25	0.097
Brazil	Cancer	-0.80	$< 10^{-7}$
USA	Cancer	-0.31	0.0381

Table S4. Correlations between health conditions and low income population in USA and Brazilian states. The Pearson coefficient r and the p-value are reported for the correlation between the percentage of people with the condition and the percentage of individuals in the bottom quintile of the income distribution in each state. Population was restricted by age depending on the condition: hypertension, diabetes and cancer are for individuals aged more than 60, fair/poor health for individuals aged less than 50.

Cover	n	n (yes)	Percentage	Percentage Mentioned	Percentage not Menti
Private health insurance	31997	18714	58.49	59.41	40.18
Medicare	31997	9263	28.95	21.35	78.23
Medicare supplement (Medigap)	31997	2263	7.07	5.0	94.59
Medicaid	31997	3225	10.08	11.07	88.52
CHIP	31997	15	0.05	0.07	99.52
Military related health care	31997	1572	4.91	3.99	95.6
Indian Health Service	31997	140	0.44	0.43	99.15
State-sponsored health plan	31997	804	2.51	2.75	96.84
Other government program	31997	368	1.15	1.18	98.41
Uninsured	31997	2767	8.65	11.15	88.44

Table S5. Distribution of health insurance coverage in the United States, based on data from the 2019 National Health Interview Survey (NHIS). The majority of individuals reported having private health insurance (59.4%), followed by Medicare (21.4%) and Medicaid (11.1%). Smaller proportions were covered by Medicare supplement plans (5.0%), military-related health care (0.1%), CHIP (0.4%), Indian Health Service (2.8%), state-sponsored or other government programs (1.2%), while 11.2% of the population reported being uninsured.

Label	n	n (yes)	Quintile	Weighted percentage (%)	CI (%)
Private health insurance	7562	1905	Bottom	25.3	(24.0, 26.6)
	5761	4954	Top	86.5	(85.4, 87.6)
Uninsured	7562	1069	Bottom	18.5	(17.3, 19.7)
	5761	123	Top	3.1	(2.4, 3.8)

Table S6. Private health insurance coverage in the USA. Distribution of private health insurance coverage and lack of insurance across household income quintiles in the USA, based on NHIS 2019. The proportion of individuals with private coverage increases sharply from 25.3% in the bottom income quintile to 86.5% in the top quintile, while the prevalence of being uninsured decreases from 18.5% to 3.1%. Percentages are reported with 95% confidence intervals.

Region	Quintile	n	n (yes)	Weighted percentage (%)	CI 95%
North	Bottom	1447	1303	89.1	(86.6, 91.6)
	Top	1448	490	32.2	(28.3, 36.1)
Northeast	Bottom	2755	2482	88.5	(86.4, 90.6)
	Top	2809	709	26.4	(23.9, 28.9)
Southeast	Bottom	2097	1705	82.6	(80.3, 84.9)
	Top	1950	225	12.3	(10.1, 14.5)
South	Bottom	936	803	88.3	(86.0, 90.6)
	Top	1166	258	22.6	(19.5, 25.7)
Midwest	Bottom	754	635	86.1	(83.1, 89.1)
	Top	1043	139	13.3	(10.7, 15.9)
Brazil	Bottom	9912	8654	86.4	(85.3, 87.5)
	Top	7243	1248	17.1	(15.7, 18.5)

Table S7. Number of respondents reporting that they received care through the Brazilian Unified Health System (SUS) in the last two weeks, the weighted percentage of such responses with corresponding 95% confidence intervals by region in Brazil and income quintile. Estimates are weighted to represent the national population.

Region	Quintile	n	n (yes)	Weighted percentage (%)	CI 95%
North	Bottom	15861	125	1.3	(1.0, 1.6)
	Top	13365	4670	38.8	(37.6, 40.0)
Northeast	Bottom	25720	236	1.3	(1.1, 1.5)
	Top	19752	9075	44.6	(43.6, 45.6)
Southeast	Bottom	15026	1128	8.6	(7.9, 9.3)
	Top	11221	8466	74.1	(72.9, 75.3)
South	Bottom	7365	445	7.9	(7.0, 8.8)
	Top	6862	4522	65.8	(64.4, 67.2)
Midwest	Bottom	7430	343	6.0	(5.2, 6.8)
	Top	6876	4324	61.6	(60.1, 63.1)
Brazil	Bottom	86086	1982	3.7	(3.5, 3.9)
	Top	47697	29374	63.9	(63.2, 64.6)

Table S8. Private health insurance coverage in Brazil. Number of respondents, number reporting that they have a private health plan or health insurance, and the weighted percentage of such responses with corresponding 95% confidence intervals by region in Brazil and income quintile. Estimates are weighted to represent the national population.

Region	Quintile	n	n (yes)	Weighted percentage (%)	CI 95%
North	Bottom	9119	8814	95.8	(95.0, 96.6)
	Top	9603	4768	45.2	(43.7, 46.7)
Northeast	Bottom	15748	15193	96.7	(96.3, 97.1)
	Top	15047	6417	43.1	(42.0, 44.2)
Southeast	Bottom	9394	8440	90.9	(90.1, 91.7)
	Top	8650	1580	20.1	(18.8, 21.4)
South	Bottom	4652	4250	91.1	(90.1, 92.1)
	Top	5385	1526	29.6	(28.0, 31.2)
Midwest	Bottom	4062	3729	91.4	(90.2, 92.6)
	Top	5066	1357	28.2	(26.6, 29.8)
Brazil	Bottom	54680	51778	94.1	(93.8, 94.4)
	Top	35727	10157	28.7	(27.9, 29.5)

Table S9. Healthcare-seeking behavior in Brazil. Number of respondents, number reporting that they usually seek care in the public health system, and the weighted percentage of such responses with corresponding 95% confidence intervals by region in Brazil and income quintile. Estimates are weighted to represent the national population.

Region	Quintile	n	Income (US$)	OOP (US$)	Income fraction (%)
Northeast	Bottom	964	7728	5316	68.8
	Top	862	143731	6530	4.5
Midwest	Bottom	1453	8170	3943	48.3
	Top	1037	143188	8061	5.6
South	Bottom	3213	7405	3484	47.0
	Top	1534	139670	6185	4.4
West	Bottom	1739	7945	3001	37.8
	Top	1297	146546	7980	5.4
USA	Bottom	7369	7725	3724	48.2
	Top	4730	142917	7101	5.0

Table S10. Out-of-pocket (OOP) expenditures in the USA. OOP as a fraction of income, by census region in the USA and income quintile. N represents the number of households in each group. The values in Income (US$) and OOP (US$) are weighted means of values per capita, while Income Fraction (%) represents the share of household income allocated to health expenses. Results show marked regional and income-based disparities: lower-income families consistently devote a higher fraction of their income to health-related OOP expenses, with the burden decreasing substantially for the top income quintile across all regions.

Region	Quintile	n	Income (R$)	OOP (R$)	Income percentage (%)
North	Bottom	2484	482	39	8.0
	Top	1141	5801	327	5.6
Northeast	Bottom	5918	480	56	11.7
	Top	2261	6010	766	12.7
Southeast	Bottom	1805	535	73	13.7
	Top	3697	7041	921	13.1
South	Bottom	583	519	73	14.0
	Top	2629	5942	550	9.3
Midwest	Bottom	784	517	73	14.1
	Top	1847	7581	831	11.0
Brazil	Bottom	11574	499	60	12.1
	Top	11575	6677	791	11.8

Table S11. Out-of-pocket (OOP) expenditures in Brazil. OOP as a fraction of income, by region in Brazil and income quintile. N represents the number of households in each group. The values in Income (R$) and OOP (R$) are weighted means of values per capita, expressed in Brazilian reais (R$), while Income Fraction (%) represents the share of household income allocated to health expenses.

Condition	Brazil: mean age bottom quintile	Brazil: mean age top quintile	USA: mean age bottom quintile	USA: mean age top quintile
Hypertension (age>60)	68.4	70.3	73.0	69.5
Diabetes (age>60)	68.4	70.3	73.0	69.5
Fair/poor health (age<50)	32.4	35.0	34.9	36.9
Obesity	38.5	48.6	49.3	52.8
Psychiatric condition	38.3	48.5	49.3	52.8
Cancer diagnosis (age>60)	68.4	70.3	73.0	69.5

Table S12. Age balance across income quintiles within each stratum. Weighted mean age (years) in the bottom and top income quintiles, within each country/condition age stratum. Age is included directly as a covariate in every regression in Tables S2–S3; this table is a complementary descriptive check showing where, if anywhere, an age imbalance between quintiles remains. Notably, for obesity and psychiatric conditions the top income quintile is on average about 10 years older than the bottom quintile in Brazil, and about 3–4 years older in the USA, underscoring the importance of the age adjustment for these two outcomes.

SUPPLEMENTARY FIGURES

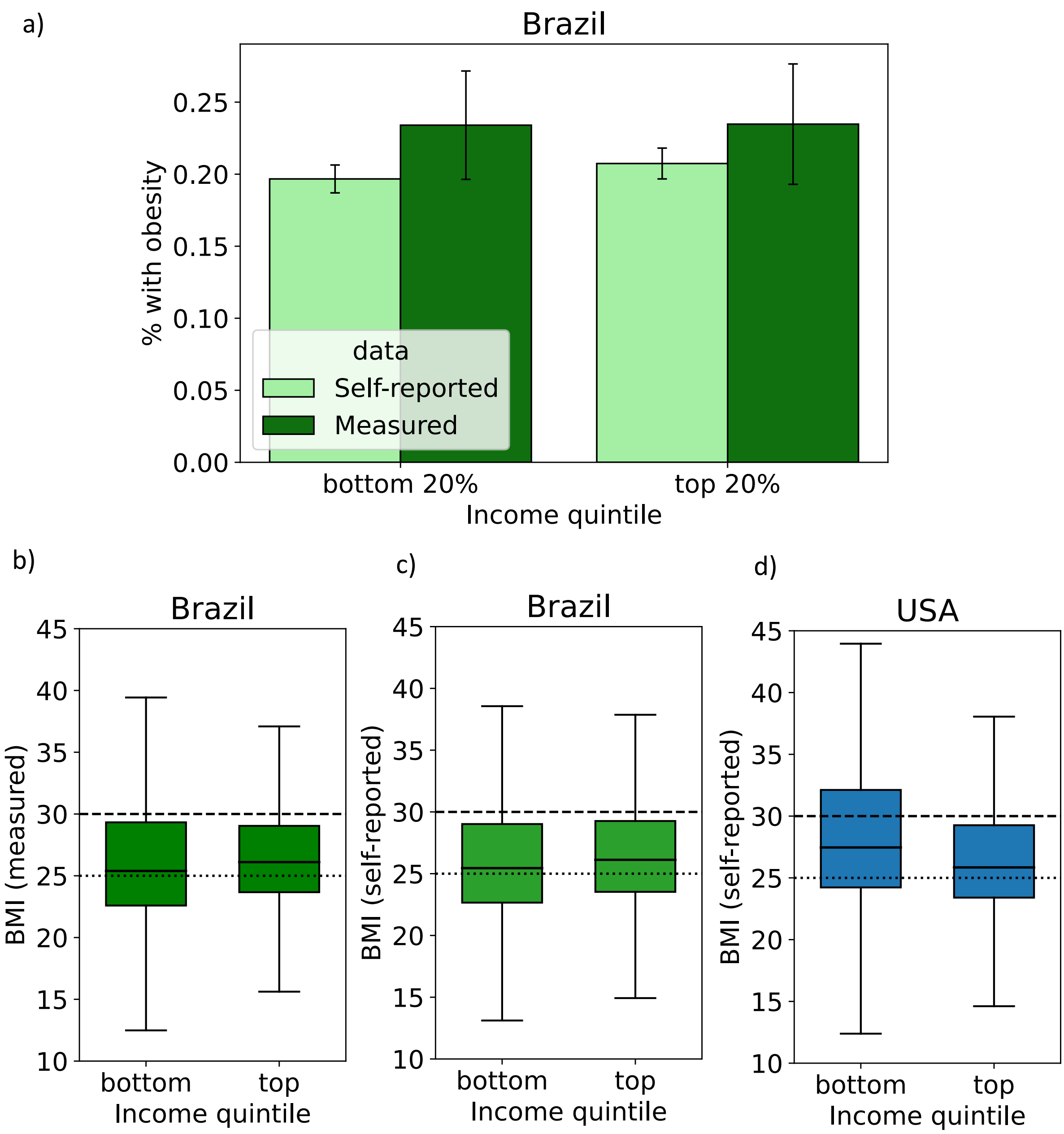

Figure S1. a) Comparison between self-reported and measured obesity for top and bottom income groups in Brazil. Boxplots of BMI for top and bottom income groups in b) Brazil (measured), c) Brazil (self-reported), and d) USA (self-reported). The dashed lines correspond to a BMI of 30, the threshold for obesity, and the dotted line to a BMI of 25, the threshold for overweight.
