# Supplementary material for: Differences in income-related health inequalities in Brazil and the USA: a comparative cross-sectional study
Source: Front Public Health. 2026 Oct 13;14:1917651. doi: 10.3389/fpubh.2026.1917651 (PMC13729126; doi:10.3389/fpubh.2026.1917651)
Supplement: Supplementary file 2 [file Data_Sheet_2.pdf]

STROBE Statement

Checklist for Cross-Sectional Studies

Manuscript: "Differences in Income-Related Health Inequalities in Brazil and the USA: A Comparative Cross-Sectional Study"

This checklist follows the standard STROBE (STrengthening the Reporting of OBservational Studies in Epidemiology) items for cross-sectional studies. For each item, the *Location* field indicates where it is addressed in the manuscript and Supplementary Material.

Title and Abstract

1(a) Indicate the study's design with a commonly used term in the title or the abstract.

Location: Title ("...A Comparative Cross-Sectional Study"); Abstract.

1(b) Provide in the abstract an informative and balanced summary of what was done and what was found.

Location: Abstract.

Introduction

2 Explain the scientific background and rationale for the investigation being reported.

Location: Introduction.

3 State specific objectives, including any prespecified hypotheses.

Location: Introduction.

Methods

4 Present key elements of study design early in the paper.

Location: Methods §2.1 (Data); Abstract.

5 Describe the setting, locations, and relevant dates, including periods of recruitment, exposure, follow-up, and data collection.

Location: Methods §2.1.

6(a) Give the eligibility criteria, and the sources and methods of selection of participants.

Location: Methods §2.1 (survey sampling frames) and §§2.2–2.3 (age-band and income-quintile restrictions per outcome).

7 Clearly define all outcomes, exposures, predictors, potential confounders, and effect modifiers; give diagnostic criteria, if applicable.

Location: Methods §2.2 (Variables), §2.4 (Statistical Analysis).

8 For each variable of interest, give sources of data and details of methods of assessment (measurement). Describe comparability of assessment methods if there is more than one group.

Location: Methods §2.2 (self-reported vs. measured BMI; self-rated health coding; income construction).

9 Describe any efforts to address potential sources of bias.

Location: Methods §2.2 (informal-income under-reporting), §2.4 (cluster-robust standard errors for complex survey design); Discussion (self-report validity, limitations).

10 Explain how the study size was arrived at.

Location: Methods §2.1 (source survey size: n = 293,726 for PNS, n = 14,410 for PSID); Supplementary Table S1 (final analytic sample size and number of events for each outcome after age-band, quintile, and complete-case restrictions).

11 Explain how quantitative variables were handled in the analyses. If applicable, describe which groupings were chosen and why.

Location: Methods §§2.2–2.3 (age bands, income quintiles, dichotomization rationale).

12(a) Describe all statistical methods, including those used to control for confounding.

Location: Methods §2.4.

12(b) Describe any methods used to examine subgroups and interactions.

Location: Methods §2.4 (country×income interaction test).

12(c) Explain how missing data were addressed.

Location: Methods §2.4 (complete-case analysis; per-model analytic sample sizes in Supplementary Table S1).

12(d) If applicable, describe analytical methods taking account of sampling strategy.

Location: Methods §2.4 (survey weights; PSU/strata clustering for Brazil; family-level clustering for the USA).

12(e) Describe any sensitivity analyses.

Location: Methods §2.4 (age-adjusted specification presented as a sensitivity analysis relative to the unadjusted model).

Results

13(a) Report the numbers of individuals at each stage of the study.

Location: Supplementary Table S1 (final analytic n and n(yes) per condition, country, and quintile). As this is a secondary analysis of two established public-use survey programs — the Pesquisa Nacional de Saúde (PNS), coordinated by IBGE, and the Panel Study of Income Dynamics (PSID), coordinated by the Institute for Social Research, University of Michigan — earlier-stage participant flow (sampling-frame construction, eligibility screening, response at first contact) is a property of those original survey designs and is documented in each survey's own technical and methodology reports.

13(b) Give reasons for non-participation at each stage.

Location: Documented in the original PNS (IBGE) and PSID (ISR) survey methodology reports, as described under item 13(a).

13(c) Consider use of a flow diagram.

Location: Not included. For this secondary analysis, the informative sample derivation — source-survey sampling frame (documented by IBGE/ISR) to final per-model analytic n — is reported directly in Supplementary Table S1 for all six outcomes and both countries.

14(a) Give characteristics of study participants and information on exposures and potential confounders.

Location: Supplementary Table S1 (prevalence by condition, country, and quintile); Supplementary Table S12 (age balance by quintile).

14(b) Indicate the number of participants with missing data for each variable of interest.

Location: Supplementary Table S1 (analytic n per model reflects the complete-case sample after exclusion for missing values, per Methods §2.4). Item-level nonresponse in the underlying survey instruments is documented by IBGE (PNS) and ISR (PSID).

14(c) Summarize follow-up time.

Location: Not applicable (cross-sectional design; no follow-up).

15 Report numbers of outcome events or summary measures.

Location: Supplementary Table S1 (n(yes) and prevalence for every condition, country, and quintile).

16(a) Give unadjusted estimates and, if applicable, confounder-adjusted estimates and their precision. Make clear which confounders were adjusted for and why they were included.

Location: Results §3.2 (crude prevalence and adjusted odds ratios reported in text); Table 1 (main text); Supplementary Tables S2–S3 (full adjusted models).

16(b) Report category boundaries when continuous variables were categorized.

Location: Methods §§2.2–2.3 (income-quintile definition; age-band boundaries).

16(c) If relevant, consider translating estimates of relative risk into absolute risk for a meaningful time period.

Location: Results §3.2 (crude prevalence percentages reported alongside every odds ratio).

17 Report other analyses done.

Location: Results §3.2 (country×income interaction test, Table 1; self-reported obesity sensitivity analysis); Results §3.3 (state-level ecological correlations).

Discussion

18 Summarize key results with reference to study objectives.

Location: Discussion, opening paragraphs.

19 Discuss limitations of the study, taking into account sources of potential bias or imprecision.

Location: Discussion, Limitations paragraph (cross-sectional design and causal inference, income vs. wealth measure, dichotomization, age-band coverage, geographic-unit comparability, obesity cross-country comparability, multiple comparisons).

20 Give a cautious overall interpretation considering objectives, limitations, multiplicity of analyses, results from similar studies, and other relevant evidence.

Location: Discussion.

21 Discuss the generalisability (external validity) of the study results.

Location: Methods §2.1 (survey sampling frames representing each national population); Discussion, Limitations paragraph (age-band restrictions per outcome; differing number of geographic units between countries).

Other Information

22 Give the source of funding and the role of the funders.

Location: Funding section.

This checklist accompanies the manuscript as Supplementary Material.
